# Supplementary material for: Spatially integrative metrics reveal hidden vulnerability of microtidal salt marshes
Source: Nat Commun. 2017 Jan 23;8:14156. doi: 10.1038/ncomms14156 (PMC5264011; doi:10.1038/ncomms14156)
Supplement: Supplementary Information — Supplementary Figures and Supplementary Table [file ncomms14156-s1.pdf]

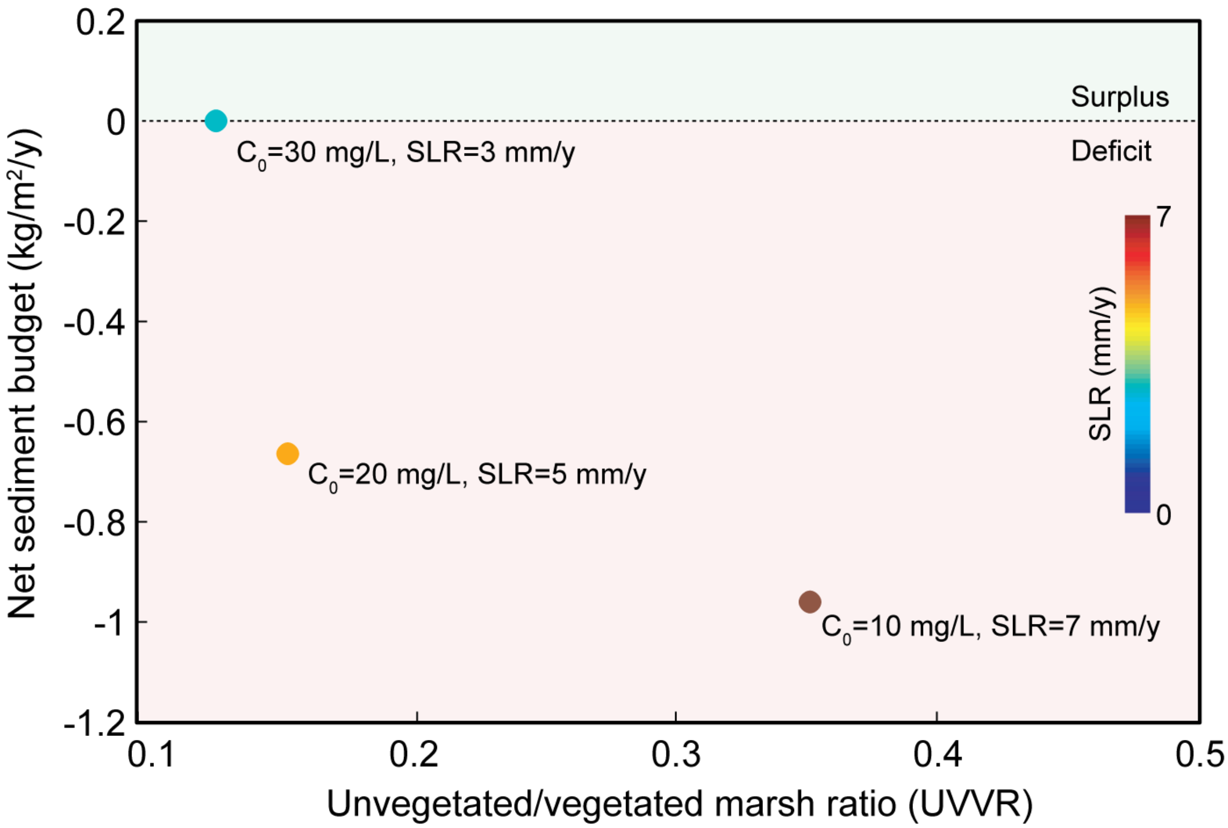

Supplementary Figure 1: Relationship between UVVR and sediment budget from idealized model

Ratio of unvegetated to vegetated marsh (UVVR) within the tidal channel drainage area versus net sediment budget (sediment flux minus supply needed to offset SLR), from idealized model simulations of Venice Lagoon. Individual points are colored to represent local sea-level rise.

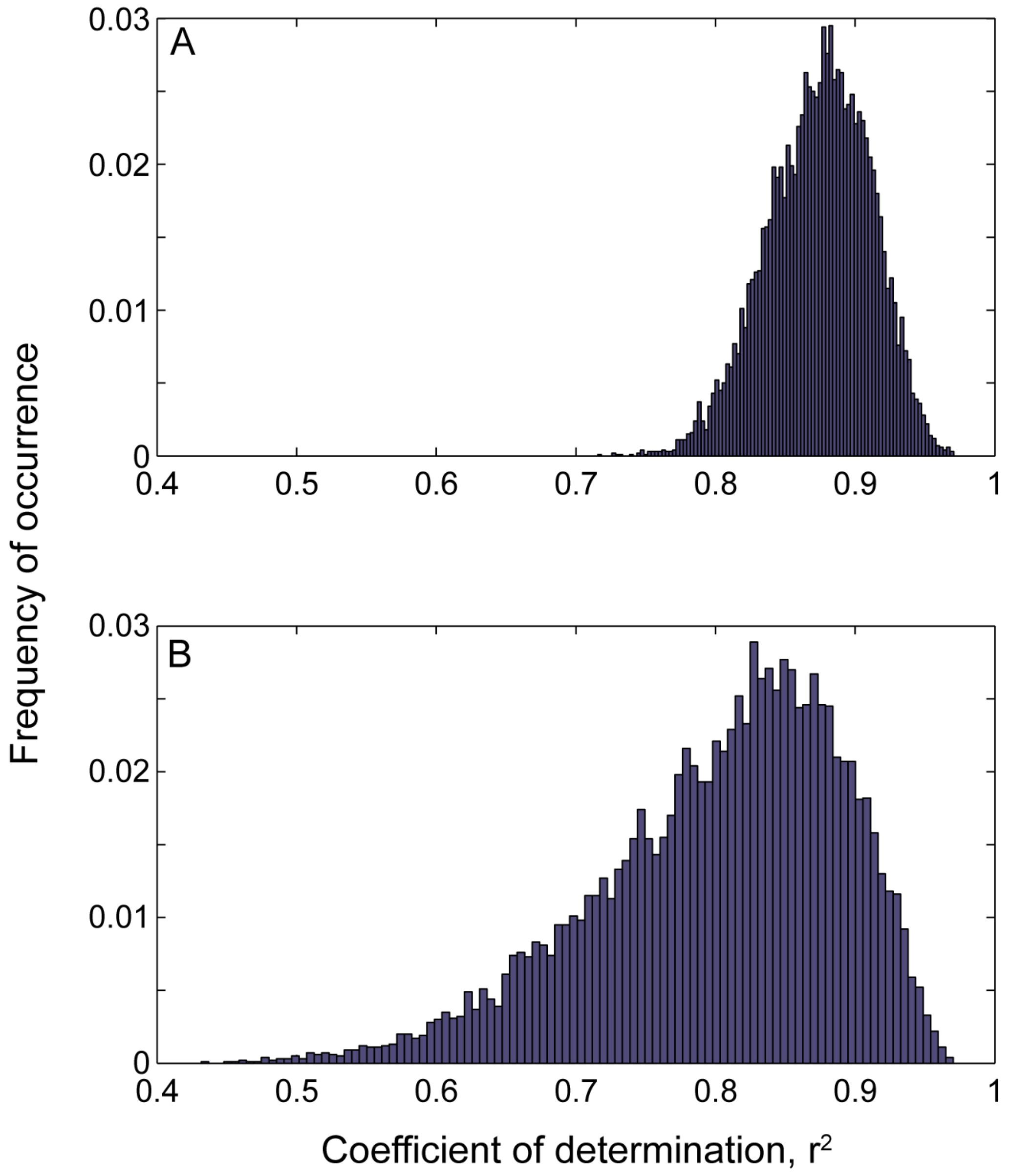

Supplementary Figure 2: Statistics for multiple realizations of correlations

Distribution of coefficients of determination (r^2) for correlations between A) UVVR and net sediment budget and B) UVVR and lifespan. Each distribution was obtained via 10000 realizations of each variable, with random errors imposed on marsh area and UVVR (panel A), and bulk density and UVVR (panel B).

Supplementary Table 1: Characteristics of sediment flux deployments used in this study.

High water events defined as prolonged events over 95th percentile of water level over entire deployment, including storm events and extreme spring tides. NR = not reported. Site PM was not surveyed intensively for cross-sectional velocity distribution due to shallow depths.

**Parameter/site**	BW	FB	SB	PM	RC	DC	OG	SC
Tidal cycles	107	147	150	152	210	402	130	112
High water events	4	7	6	5	5	14	4	NR
Maximum wind speed (m/s)	13	13	14	10	12	12	9	NR
Cross-sectional velocity surveys	129	226	63, 93	N/A	254	240	222	Continuous over 6 h
Topographic Controls	Uplands	None	Hardened structures	None	Upland	Upland	Upland, hardened structures	Hardened structures
